# Amino-Acid Characteristics in Protein Native State Structures

**DOI:** 10.3390/biom14070805

**Published:** 2024-07-07

**Authors:** Tatjana Škrbić, Achille Giacometti, Trinh X. Hoang, Amos Maritan, Jayanth R. Banavar

**Affiliations:** 1 Department of Molecular Sciences and Nanosystems, Ca’ Foscari University of Venice, Campus Scientifico, Via Torino 155, 30170 Venice Mestre, Italy; achille@unive.it; 2 Department of Physics and Institute for Fundamental Science, University of Oregon, Eugene, OR 97403, USA; banavar@uoregon.edu; 3 European Centre for Living Technology (ECLT), Ca’ Bottacin, Dorsoduro 3911, Calle Crosera, 30123 Venice, Italy; 4 Institute of Physics, Vietnam Academy of Science and Technology, 10 DaoTan, Ba Dinh, Hanoi 11108, Vietnam; txhoang@iop.vast.vn; 5 Department of Physics and Astronomy, University of Padua, Via Marzolo 8, 35131 Padua, Italy; amos.maritan@unipd.it

**Keywords:** local Frenet frame, amino-acid classes, side-chain protrusion, pre-sculpted landscape

## Abstract

The molecular machines of life, proteins, are made up of twenty kinds of amino acids, each with distinctive side chains. We present a geometrical analysis of the protrusion statistics of side chains in more than 4000 high-resolution protein structures. We employ a coarse-grained representation of the protein backbone viewed as a linear chain of C_α_ atoms and consider just the heavy atoms of the side chains. We study the large variety of behaviors of the amino acids based on both rudimentary structural chemistry as well as geometry. Our geometrical analysis uses a backbone Frenet coordinate system for the common study of all amino acids. Our analysis underscores the richness of the repertoire of amino acids that is available to nature to design protein sequences that fit within the putative native state folds.

## 1. Introduction

Proteins are relatively short linear chains of amino acids with a common backbone. There are twenty types of naturally occurring amino acids, each possessing a distinct side chain attached to the main chain protein backbone [1,2,3,4]. The complexity of the protein problem stems from the myriad degrees of freedom. A protein is surrounded by water molecules within the cell. Each of the twenty side chains has its own chemical properties and geometry. Despite the complexity, small globular proteins share a great deal of properties because of their common backbone. They fold rapidly and reproducibly into their respective unique native state structures [5]. Protein native state structures are modular and comprise secondary structure building blocks: topologically one-dimensional α-helices and almost planar parallel and antiparallel β-sheets. Hydrogen bonds provide support to the building blocks [6,7]. A typical protein of modest length may have around a dozen building block segments of either a helix or a strand. The total number of distinct native fold topologies ought then to be of the order of several thousand [8,9,10,11] estimated as the product of 2^12^ (corresponding to the number of distinct ways in which one can choose the segments) and the distinct turn topologies that connect them. Furthermore, the native state folds are evolutionarily conserved [12,13]. This surprising simplicity present in the complex protein problem can be rationalized through the notion of a free energy landscape of proteins sculpted by the common backbone of all proteins [14,15].

**Figure 1:**
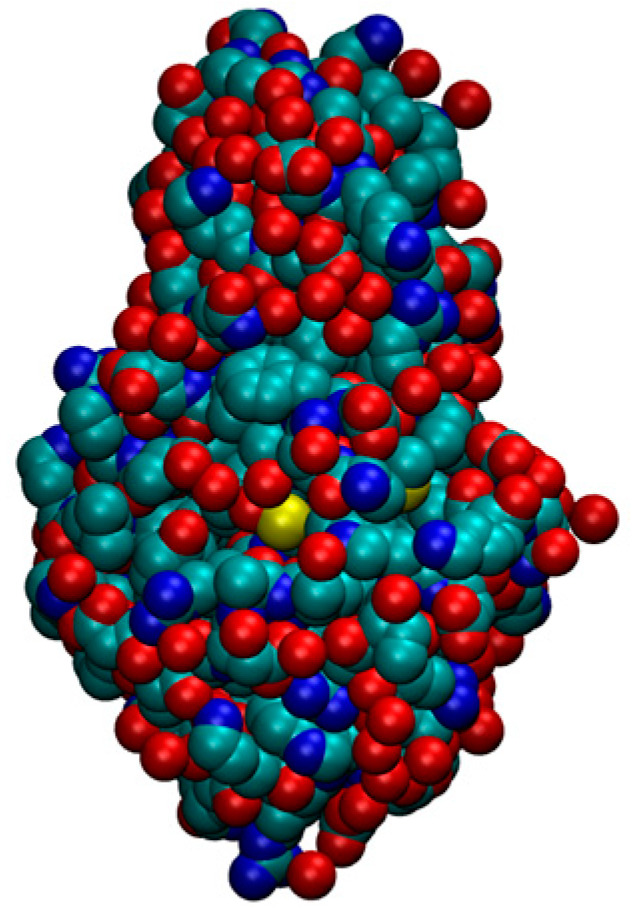
Native state of bacteriophage T4 lysozyme (PDB code: 2LZM) in the CPK representation [23,24] in which all heavy atoms of the protein backbone and its side chains are represented as spheres with radii proportional to their respective van der Waals atomic radii. Color code: carbon (cyan), oxygen (red), nitrogen (blue), and sulfur (yellow). The side chains in the protein interior are very well packed.

The side chains play a critical role in the selection process in two crucial ways. First, the chemistry of the interacting side chains [16,17] must be harmonious [18,19,20,21], maximizing favorable interactions (including water-mediated hydrophobic, van der Waals, electrostatic, and hydrogen bonding interactions). The net result is to create a protein hydrophobic core shielded from the surrounding water molecules, thereby ensuring the stability and compactness of the protein native structure. Second, the side chains must fill the space in the interior of the protein, packing tightly against each other, maximizing favorable self-interactions in the hydrophobic interior, and minimizing empty space [22,23,24] (see Figure 1). Interestingly, even in toy chain models [25,26,27], adding side chain spheres to the canonical tangent sphere model and permitting adjoining spheres to overlap, destabilizes the disordered compact globular phase and results in novel structured phases with effectively reduced dimensionalities.

The specific arrangement of side chains within the protein interior has been studied for several decades [18,19,20,21,22,23,24,25,26,27,28,29,30,31,32,33,34,35,36,37,38,39,40,41,42,43] and is determined by at least two factors. The first is the primary protein sequence of amino acids that can grossly be classified as being hydrophobic (non-polar residues mainly buried in the protein interior and forming its hydrophobic core), hydrophilic (polar or charged residues that readily interact with water molecules and tend to be positioned at the protein surface), or neutral (somewhere between the two categories) [28]. The second is that the overall folded geometry ought to provide an optimal, best possible fit to the sequence. The orientation of the side chain is flexible and the set of specific conformations and/or orientations that are statistically significant constitute the so-called side chain rotamers [29,30,31,32,33,34]. There could also be an entropic cost associated with freezing a side chain into a particular rotamer conformation, which may be more relevant in the denatured state.

Here, we adopt a simplified coarse-grained description. We view a protein as a chain of C_α_ atoms. Our approach then consists in determining the locations and orientations of the protruding side chain atoms. Because of the imperative need to fill space in the interior while assiduously avoiding steric clashes, our focus is on the heavy atom protruding furthest from the corresponding C_α_ atom. The novelty of our work is the characterization of the geometry of this protrusion in a universal coordinate frame relative to the portion of protein backbone corresponding to the given amino acid, which enables us to determine both the average side chain behavior as well as the specific behavior of distinct amino acids. We do this through a detailed analysis of over 4000 high-precision native state structures. We alert the reader that the results we present here are but the first step on a longer journey. With the availability of the results presented here, we wish to set the stage for the more important step of understanding the role of side chains in tertiary structure assembly.

Our analysis of side chain protrusion in the native state folds of proteins can be useful for understanding the geometry of protein native state structures and their stability. In Section 3.4, we illustrate this with a biological example of fold switching [44,45,46,47,48], where a very small number of mutations can result in a fold switch. We show that the geometry of protrusion of amino acids plays a critical role in determining the quality of fit or misfit of the side chains in the protein interior, which, in turn, impacts on the viability of a fold.

## 2. Materials and Methods

### 2.1. Local Frenet Coordinate System of an Amino Acid

**Figure 2:**
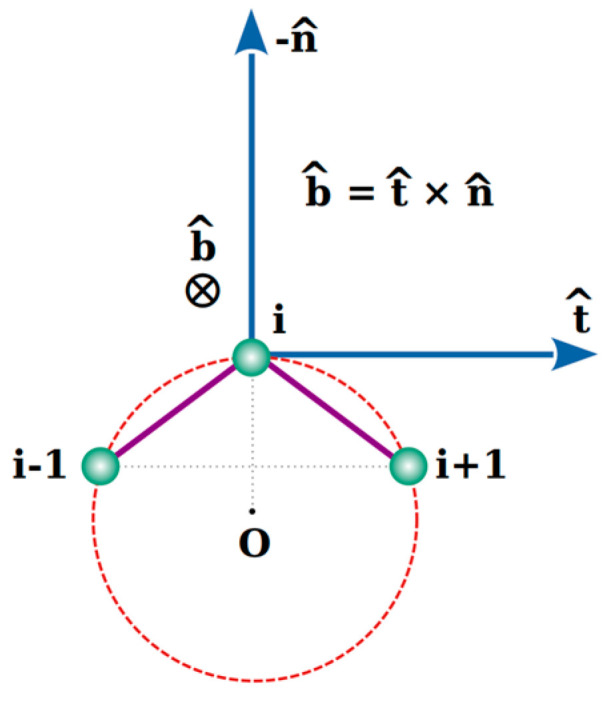
Local Frenet frame of the amino acid i. The three consecutive C_α_ atoms are at points i − 1, i, and i + 1 and lie in the plane of the paper. The point O is at the center of a circle passing through them. Please see text for a description of the orthonormal basis set.

We view a protein backbone as a chain of discrete points on which the consecutive C_α_ atoms are located. We account for all heavy atoms of the side chains when determining the maximally protruding side chain atom from the protein backbone (thus excluding hydrogen atoms from our analysis), because only heavy side chain atoms effectively contribute to the definition of side chain rotamers [29,30,31,32,33,34]. The maximally protruding atom of a side chain is the farthest heavy atom from the corresponding C_α_ atom and at a distance that we call Rmax. To characterize the orientation of this maximally protruding side chain atom, we employ a Frenet coordinate system [49] local to the portion of the backbone to which the side chain belongs. For the i-th amino acid in question, the origin of its local Frenet frame is located at the i-th C_α_ atom. The orthonormal set of axes are the tangent **t**, anti-normal **an** = **−n**, and binormal **b**. These basis vectors are defined from the positions of three consecutive C_α_ atoms associated with residues i − 1, i, and i + 1, as shown in Figure 2.

About 99.7% of the C_α_-C_α_ pseudo-bond lengths in proteins are, to a very good approximation, equal to 3.81 Å [50], corresponding to the prevalent *trans* isomeric conformation of a peptide backbone group, where the two neighboring C_α_ atoms along the chain are on opposite sides of the peptide bond with the third Ramachandran angle of ω close to 180°. However, the remaining ~0.3% of protein bonds are shorter, having a length around ~2.95 Å [50] and correspond to the so-called *cis* conformation of a backbone [51], in which the two consecutive C_α_ atoms are placed on the same side of the connecting peptide bond, when the third Ramachandran angle is ω ~ 0°. We define a local Frenet frame of a given amino acid in a manner that is robust to variations in the bond lengths. First, independent of the bond lengths, we draw a circle passing through points i − 1, i, and i+1 and determine its center and the radius. The direction of the anti-normal (negative normal direction) **an** = **−n** is along the straight line joining the center of the circle to the C_α_ atom. The tangent vector **t** points along the direction (i − 1, i + 1). Both the tangent and normal vectors are in the plane of the paper in Figure 2. The binormal vector **b** is found as a cross-product of the unit vectors **t** × **n** and is perpendicular and into the plane of the paper (see Figure 2). The Frenet frame is well defined at all but the end sites of a protein chain and serves as a convenient reference frame for studying the side chain protrusion of all amino acids in the native state structures. We characterize the orientation of the maximally protruding heavy atom of the side chain from the C_α_ atom by means of three projections, along the unit vectors **t**, **b**, and **−n**, in the corresponding local Frenet system.

### 2.2. Curation and Data Analysis

Our protein data set consists of 4366 globular protein structures from the PDB, a subset of Richardsons’ Top 8000 set [52] of high-resolution, quality-filtered protein chains (resolution < 2 Å, 70% PDB homology level), that we further distilled out to exclude structures with missing backbone and side chain atoms, as well as amyloid-like structures. The program DSSP (CMBI version 2.0) [53] was used to determine the context, in an α-helix, in a β-strand or elsewhere, for each protein residue in each of the native state structures.

Our data set comprises a total of 959,691 residues (883,407 non-glycine and 76,284 glycine amino acids) in the native state structures of more than 4000 proteins. Their abundances and relative frequencies, in order of decreasing prevalence, in our data set, are shown in Table 1.

## 3. Results

### 3.1. The Orientation of Amino Acids in Globular Proteins

For each amino acid in our data set of proteins, we determine a protrusion vector in the Frenet frame which connects a C_α_ atom to the maximally protruding heavy atom in its side chain. By maximally protruding, we mean the heavy atom that is the farthest away from the C_α_ atom. This provides a rough idea of the spatial extent and the relevant direction of the side chain of the residue. The presence of rotamers in the native structures of proteins immediately implies that not all amino acids of a given type will have the same protrusion vector. Our analysis aims to determine the statistics of protrusion of all side chains and of the side chains of individual amino acid types.

Our results on the protrusion for all amino acids in our data set, as well as for the nineteen amino acids separately are summarized in Table S1 in Supplementary Information (SI). We begin by averaging the protrusion vectors of all amino acids in our data set to determine an average protrusion vector, characterized by its magnitude, and the components of the normalized unit average protrusion vector along the three Frenet axes (the squares of these components add up to 1). With the notable exception of proline, the average protrusion vector lies predominantly in the (anti-normal–binormal) plane with a relatively small component in the tangent direction (see Table S1). More specifically, the resulting protrusion vector averaged over all amino acids in our data set forms angles of 26.71°, 92.44°, and 116.58°, with the anti-normal, tangent, and binormal vectors, respectively. Interestingly, amino acids predominantly point close to the anti-normal direction, thus avoiding the protein backbone. Additionally, the magnitude of the mean protrusion vector of all amino acids is found to be 3.81 Å matching the distance between consecutive C_α_ atoms along the chain. This equality of two characteristic lengths in proteins, one along the protein backbone and the second approximately perpendicular to it, is noteworthy. Table S1 in Supplementary Information also presents analogous data for the nineteen amino acids possessing heavy atoms in their side chains. This excludes glycine, which has none.

**Figure 3:**
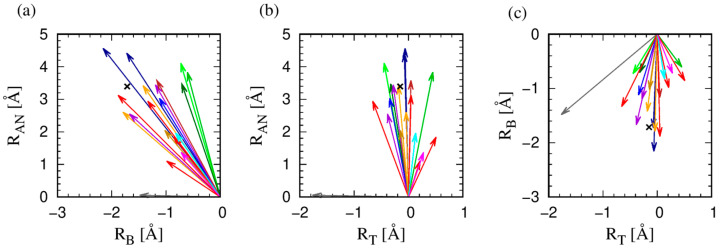
Two-dimensional projections of the mean maximal protrusion of nineteen amino acids in more than 4000 high-resolution structures of globular proteins. For ease of visualization, we show three two-dimensional views: (**a**) in the anti-normal–binormal plane; (**b**) in the anti-normal–tangent plane; and (**c**) in the binormal–tangent plane. The color code of the protrusion vectors follows that employed in Table 2. The black X symbols in all the three panels denote the end point of the projection of the mean protrusion vector calculated for all amino acids in our data set into the corresponding plane.

To obtain a measure of the spread of the data around the average value for a given amino acid, we use two measures. The first is a ratio of the magnitude of the average protrusion vector to the average protrusion distance (measured with no regard to the varying directions), which we denote as R_eff_/<R_max_> in Table S1. We also take an average of the dot product of the individual protrusion vectors with the average protrusion vector for each amino acid and denote it as ⟨cos θ⟩ (see Table S1). Note that the two independent estimates of the spread defined in this way are in excellent accord with each other. We note that the largest spread is displayed by amino acids with a ring structure (HIS, PHE, TRP, and TYR), followed by long linear chains (ARG, GLN, GLU, and LYS). For the gallery of the nineteen amino acid types, see Figure 3.

Figure 3 depicts the vectors of the mean protrusion of the nineteen amino acids in the local Frenet frame. The magnitude of the vector is R_eff_. The figure depicts three two-dimensional views. The protrusion of the side chains is dominantly in the negative binormal–negative normal plane. Even a cursory look at Figure 3 shows that PRO (gray, almost horizontal arrow in (a) and (b)) is an outlier. PRO has a large projection in the tangent direction (that is along the backbone direction) due to its peculiar geometry that reaches back to the protein backbone. Leaving aside proline, we note (see Figure 3a,b) that the projection along the anti-normal direction spans the range of 3.5 Å between 1.1 Å (ALA, red) and 4.6 Å (ARG, dark blue). For the binormal, the values range over a smaller interval from −2.2 Å (ARG, dark blue arrow) to −0.6 Å (TRP, green arrow). Finally, along the tangent (see Figure 3b,c), the values of the projections range from −0.7 Å (ILE, again red) to 0.5 Å (VAL, another red). Let us take a closer look at Figure 3a and the directions in which the mean vectors for a given amino acid type protrude in this plane.

**Figure 4:**
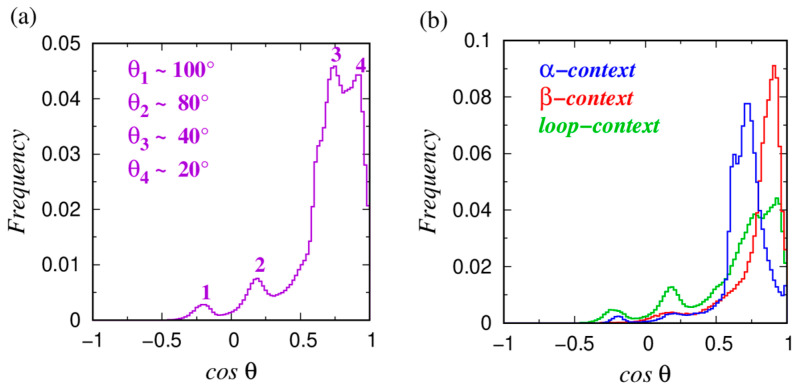
(**a**) Probability distribution of the projections (cos θ values) of the maximally protruding directions of amino-acid side chains along the anti-normal directions of their respective local Frenet frames of ~900,000 non-glycine residues in more than 4000 high-resolution structures of globular proteins. (**b**) Probability distribution of the cos θ values for the three subsets of all consecutive triplets of C_α_ atoms belonging to ‘α’-helical segments (red histogram), to ‘β’-strands (blue histogram), and those for which the consecutive triplets of C_α_ atoms are in protein loops.

**Figure 5:**
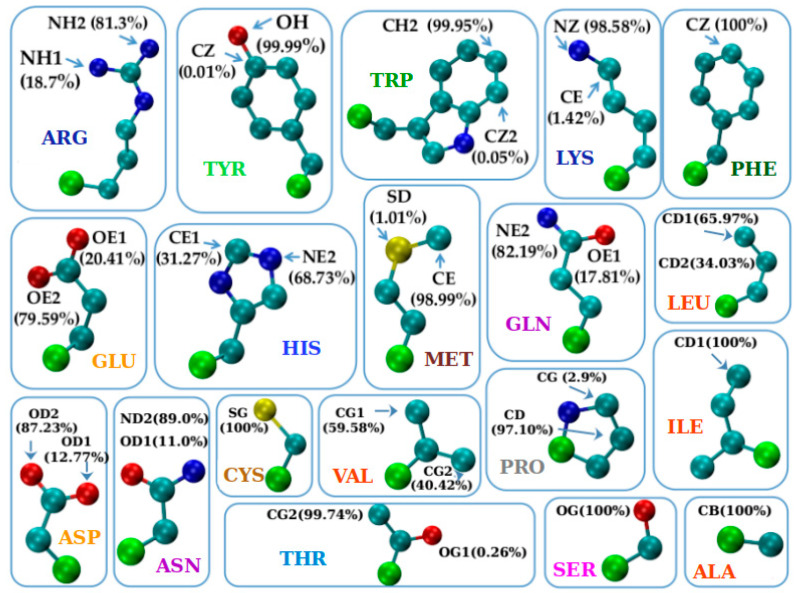
Gallery of nineteen amino acids (with glycine excluded). Three-letter amino acid codes are used. For each amino acid, the maximally protruding atom along with the frequency with which it occurs is shown. The color code of the atoms is: carbon C_α_ in green, carbon C atoms other than C_α_ in turquoise, oxygen O atoms in red, nitrogen N atoms in dark blue, and sulfur S atoms in yellow. Carbon C_α_ atoms (green spheres) are artificially represented as spheres with slightly larger radius than the rest of C atoms (cyan spheres) to enhance visibility. The measure of the degree of protrusion of a given side chain atom with respect to the backbone was defined to be the distance of the atom from the corresponding C_α_ atom. The color code of the amino-acid labels follows that in Table 2. We note that here we have adopted atom names as assigned in the PDB file, and this makes the branching numbers assigned for identical atoms spurious. NH1 and NH2 atoms in LYS; OE1 and OE2 atoms in GLU; and OD1 and OD2 atoms in ASP are indistinguishable. Nevertheless, we follow the atom nomenclature of the PDB files.

Figure 3 shows that, after PRO, ALA (red) is the next outlier. ALA with only one C_β_ carbon atom in its side chain, bonded directly to the C_α_ atom, has a highly constrained geometry of protrusion due to sp^3^ hybridization of the C_α_ atom. ALA is followed by ASP (orange) and ASN (purple) sharing essentially the same geometry. Figure 4 shows that they share the same geometrical shape, the difference being that one oxygen atom in ASP is converted to nitrogen in the case of ASN. On the other side in Figure 3a, the aromatic trio, PHE (dark green), TYR (light green), and TRP (green) form the largest angles with the binormal direction (and the smallest angles with the anti-normal direction), while sharing very similar directions. They are thus, among all amino acids, on average, pointing the most away from the backbone. On the other hand, TRP is unique in that it has a ‘double ring’ for its side chain (see Figure 5), and this makes its full protrusion geometry quite distinct (see Section 3.3).

We have also studied the variations of Figure 3 within an individual amino acid and we find the striking result that, in terms of the direction of protrusion (not magnitude), three pairs of geometrical twins show similar behaviors within a pair: (ASN and ASP); (GLN and GLU); and (PHE and TYR). Even in cases when the mean poking for an amino acid in the tangent direction is small, there are large fluctuations especially when the side chains are large in size (PHE, TRP, TYR rings and ARG, LYS linear topology).

To illustrate the sensitivity of the geometry of amino acid protrusion on its local environment (‘α’-helical, ‘β’-sheet or ‘loop’), we show, in Figure 4a, the distributions of the projections of the directions of the maximal protrusion of all ~900,000 non-glycine amino acids in our data set along the anti-normal directions of their respective local Frenet frames. For the mean values ⟨cos θ⟩ for each amino acid type, please consult Table S1 in Supplementary Information. Figure 4b shows the frequency distributions only for those amino acids that are embedded in ‘α’-helical, ‘β’-sheet, or ‘loop’ environments. They demonstrate the origins of the peaks marked ‘1’ to ‘4’ in Figure 4a. The peaks dubbed ‘3’ and ‘4’ arise from the ‘α’-helical and ‘β’-strand contexts, respectively. Interestingly, both peaks ‘1’ and ‘2’ originate primarily from the proline amino acid that is prevalently found in protein loops (see Table 1). We conclude that although we do observe a correlation between the local geometry of a protein backbone (secondary structure propensity) and the protrusion geometry of a side chain, the corresponding distributions are quite broad. Additionally, most amino acid types do not show a sharp selectivity in their secondary structure propensity (see Table 1).

### 3.2. The Protruder Atom Type and Amino Acid Groupings

Figure 5 indicates, for each of the nineteen amino acids (but glycine (GLY) that does not possess any heavy atoms), the atom that protrudes the most along with the percentage of time it does. We note that in most cases there is prevalently only one such atom (~90% or more) and this is the case for the thirteen amino acids: ALA, ASN, ASP, CYS, ILE, LYS, MET, SER, THR, TYR, TRP, PHE, and PRO. For the remaining six amino acids: ARG, GLN, GLU, HIS, LEU, and VAL there were two viable candidate atoms. We note that both hydrophilic and hydrophobic residues are present in both these classes showing that this result is largely chemistry independent.

Based on Figure 5, we now proceed to a coarse graining of the amino acids into similar groups. The combination of rudimentary structural chemistry and protrusion geometry allows us to crudely divide our amino acids into 14 groups. Glycine is a group by itself because it has no side chain heavy atoms. Likewise, proline is special because it has a ring that connects back to the backbone. The rest of the amino acids can be grouped together based on the topology of the side chain (linear or ring) and the identities of the non-carbon atoms in the side chain and the most protruding one. This yields one group with 4 amino acids and two groups with 2 amino acids each and twelve singlet groups in all. Interestingly, the 11 groups in the IMGT classification [54] result from a partial merger of our 14 groups: Group VI (ARG, LYS) with VII (HIS); Group X (SER) with XI (THR); and Group XII (CYS) with XIII (MET). Amino acids (ARG, LYS, HIS) form the so-called ‘basic’ IMGT group, composed of all positively charged amino acids among the nineteen, while (SER, THR) constitute the ‘hydroxylic’ IMGT group of polar amino acids that contain the -OH group. Finally, (CYS, MET) form the so-called ‘sulfur-containing’ IMGT group, as the only two amino acids that contain a sulfur atom. We now turn to a careful analysis of the geometry of protrusion of the side chains.

### 3.3. The Geometry of Amino-Acid Protrusion

We have observed that the mean protrusion vector calculated over all amino acids lies predominantly in the anti-normal–binormal plane of the corresponding local Frenet frames (see Table S1 in Supplementary Information). This information allows us to considerably simplify our analysis and concentrate on the protrusion behavior in this plane. To this end, we define ɛ as the angle made by the projection of an individual amino acid in the anti-normal–binormal plane with the anti-normal direction. For each of the nineteen amino acids (except for glycine, which has no heavy atoms in its side chain), we measure the distribution of ɛ. The mean, the modal value(s) (there are sometimes multiple modes), and the standard deviations are shown in Table 3. We have carried out the calculations based on the context (helix α, strand β, or loop) of the amino acids. The lessons learned are the following:

- PRO due to its distinct geometry of a ring that reconnects to the protein backbone, has characteristic ɛ values that are close to or even larger than 90°. This context-independent result reflects the fact that PRO dominantly protrudes in the binormal-tangent plane unlike all the other amino acids (see Table S1 in Supplementary Information). PRO forms the singlet ‘neutral aliphatic’ group in the IMGT classification [54] and is our singlet Group I (see Table 2);
- ALA, ILE, LEU, and VAL have qualitatively similar behaviors. For both α and β contexts, one mode strongly dominates, while in the loop context, the behavior is a combination of the modes in the α and β contexts. (ALA, ILE, LEU, VAL) form the ‘hydrophobic aliphatic’ IMGT group [54] and coincides with our Group II (see Table 2);
- PHE and TYR share very similar behavior, with only one mode present in each of the contexts and all of them ~0°, meaning that these amino acids with aromatic rings protrude predominantly along the anti-normal direction. PHE is a singlet ‘hydrophobic, aromatic, with no hydrogen donor’ and TYR a singlet ‘neutral, aromatic, with both hydrogen donor and acceptor’ group in the IMGT classification [54], We denote them as singlet groups as well, Group III and Group V (see Table 2);
- TRP is the unique amino acid with the ‘double ring’ structure (composed of a six-atom ring and a five-atom ring, sharing one side, see Figure 5) and, contrary to all other amino acids, has an ɛ angle α-mode smaller than the ɛ angle β-mode. TRP forms the singlet ‘hydrophobic, aromatic, with hydrogen donor’ IMGT group [54] and is our singlet Group IV (see Table 2);
- ARG, LYS, and HIS, the three positively charged amino acids forming the ‘basic’ group in IMGT classification [54]. They all exhibit a ~0° β-mode, but quite different α-modes. For ARG, there are two α-modes, presumably due to the ‘double tip’ branch formed by two symmetrically placed nitrogen atoms at its end (see Figure 5). In our classification, ARG and LYS fall into Group VI, while HIS forms the singlet Group VII, due to its different topology (see Table 2);
- ASP and ASN, on one hand, and GLU and GLN, on the other, have very similar ɛ angle profiles, so they can be dubbed geometrical twins. From Figure 5, we see that this is due to the identical shape for the two corresponding pairs, with the difference that for ASP and GLU the ‘double tip’ in the amino acid ending is made up of two oxygen atoms, while for the ASN and GLN, the double tip is composed of one oxygen and one nitrogen atom. In the IMGT categorization [54], ASP and GLU constitute the ‘acidic’ group, while ASN and GLN form the ‘amide’ group. In our classification, these pairs of amino acids form Group VIII and Group IX, respectively (see Table 2);
- SER and THR constitute the ‘hydroxylic’ group in the IMGT classification [54] and have decisively different protrusion geometries, with SER most notably (and distinctively from all other amino acids) displaying the most complex ɛ profile, with three α-modes, two β-modes, as well as two loop-modes. SER is thus the champion of versatility with multiple sharp modes in all environments that is surprising because of its relatively small size. For 60% of the time, SER is found in loops. In our grouping, SER and THR form two singlet groups, Group X and Group XI, respectively (see Table 2);
- CYS and MET, placed in the ‘sulfur-containing’ group in the IMGT classification [54], have different protrusion geometries. SER has a non-zero α-mode and zero β- and loop-modes; while MET with all three zero-modes, seems more compatible geometry-wise with the aromatic duo PHE and TYR. In our grouping, CYS and MET are in two singlet groups, Group XII and Group XIII (see Table 2);
- There are three amino acids, ARG, GLN, and GLU with two dominant α-modes, that could be due to their considerable length and the ‘double tip’ shape in the amino acid ending. For GLN, this is also reflected in the double peak in the distribution of the magnitude of the maximal protrusion R_max_ (see Figure 5), while for ARG, R_max_ has a very broad distribution, so that no well-defined peaks could be identified.
- Finally, GLY (with no heavy side chain atoms) is our singlet Group XIV and it belongs to the ‘very small, neutral aliphatic’ singlet group in the IMGT classification [54].

**Figure 6:**
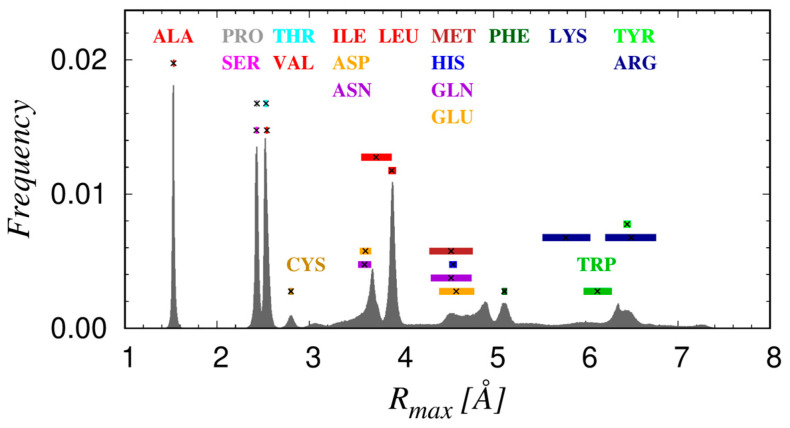
Histogram of the maximal protrusion R_max_ of amino acids in more than 4000 high-resolution structures of globular proteins. The 19 amino acids (with glycine being excluded, having no heavy side chain atoms) are denoted with a three-letter amino acid code and are colored according to the amino acid classification summarized in Table 2. The mean values of R_max_ for each of the amino acids are shown as black X symbols, while the colored rectangles have a width that corresponds to the standard deviation.

Finally, we have studied the distribution of the values of the maximal protrusion R_max_ for each of the 19 amino acids shown in Figure 6. The observed peaks in this distribution can be readily assigned to specific amino acids because of their non-overlapping mean values and their relatively sharp widths. Additionally, we can conveniently divide the observed range of R_max_ into three distinct classes: (1) small with R_max_ < 3 Å, comprising ALA, CYS, PRO, SER, and VAL; (2) medium R_max_ ~ (3–5) Å, composed of ASN, ASP, GLN, GLU, HIS, ILE, LEU, and MET; and (3) large with R_max_ > 5 Å, containing ARG, LYS, PHE, TRP, and TYR.

**Figure 7:**
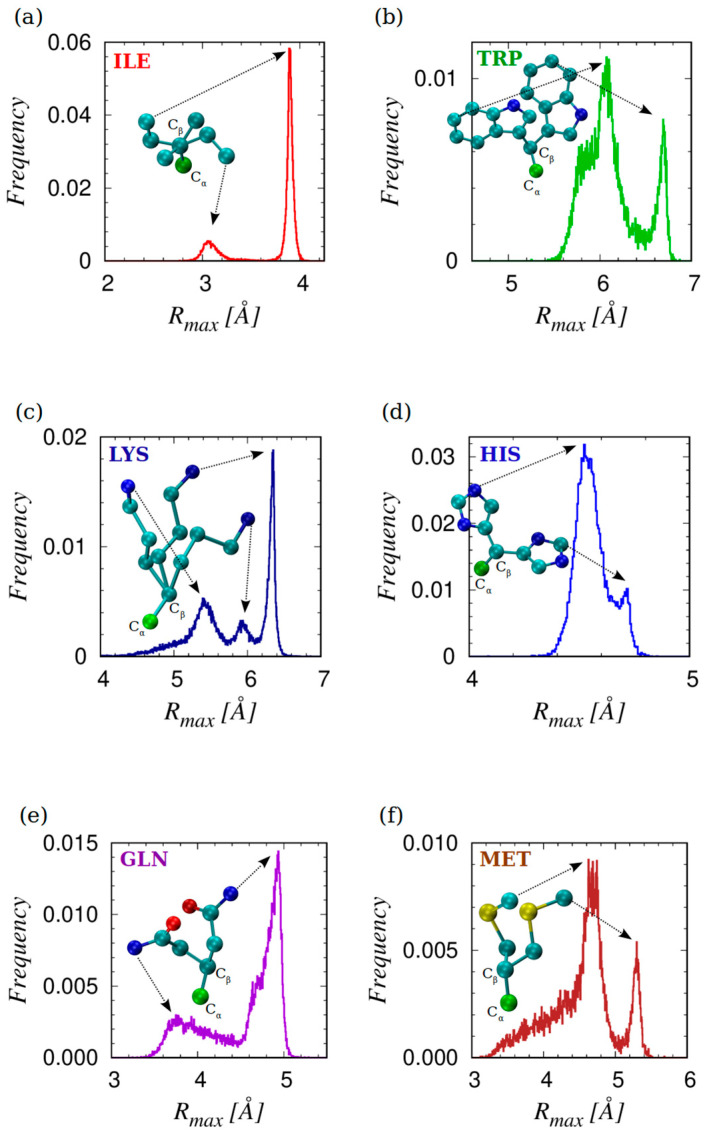
Sketches of the histograms of R_max_ and conformations associated with the multiple modes for six amino acids: (**a**) ILE; (**b**) TRP; (**c**) LYS; (**d**) HIS; (**e**) GLN; and (**f**) MET. For each set of rotamers, the C_α_ and C_β_ atoms are superimposed to better visualize the distinction between the conformations. The arrows link the maximally protruding atom to the corresponding mode in the R_max_ frequency distribution. The atoms are color coded: carbon C_α_ in green, carbon C atoms other than C_α_ in turquoise, oxygen O atoms in red, nitrogen N atoms in blue, and sulfur S atoms in yellow.

We find that there is no significant dependence of R_max_ on the context. However, there are a few cases in which the distributions clearly show resolved multiple peaks. These cases are shown in Figure 7 along with typical conformations that yield the distinct values of R_max_. Except for six amino acids, ILE, GLU, HIS, LYS, and MET (which exhibit more than one peak) and ARG (which has a very broad distribution), the amino acids exhibit one sharp mode in the R_max_ distribution. The most protruding atom in ILE, LYS, MET, and TRP does not depend on the mode, carbon for ILE, MET and TRP and nitrogen for LYS (see Figure 5 for the nomenclature of the atoms in the side chains). For HIS and GLN, the situation is more varied. GLN’s lower peak of ~3.8 Å in ~70% of cases result from nitrogen atom protrusion while the remaining results from the oxygen atom (see Figure 5). HIS has two close but well-resolved peaks. The more dominant one at ~4.5 Å is caused in ~80% of cases by the nitrogen atom protruding the most, while in ~20% of cases the protrude is a carbon atom. In addition, the considerably smaller mode at ~4.7 Å is, in more than ~90% of cases, caused by the maximal protrusion of a carbon atom (see Figure 5).

### 3.4. The Biology of Amino Acid Protrusion

There is compelling evidence that even a single mutation of a critically important amino acid can result in fold switching [44,45,46,47,48]. Such switching can arise when there is an incompatibility of the chemistry of amino acid interactions. The geometry of protrusion may also be implicated in fold switching because of putative overlap or the undesirable opening of empty space between interacting amino acids leading to non-optimal packing. Interestingly, even the stability of a given fold can also be affected by the imperfect fit of amino acid geometries. This is where our geometrical analysis can become relevant.

In important experimental work [47], it was shown that a conformational switch from α+4β to 3α topology occurs via a single amino acid substitution, that confers distinct functionalities to the sequence. The α+4β fold is adopted by Protein G, the immunoglobin (IgG) binding protein, a cell surface protein used for purifying antibodies. An almost identical sequence (with a single mutation) adopts a 3α fold, which allows binding of human serum albumin (HSA), a major contaminant of antibody sources. Both mutants are marginally stable with unfolding temperatures of around 36 °C. Just one additional mutation results in the three-helix bundle with a significantly increased stability reflected in an unfolding temperature of 50 °C [47].

The amino acid substitutions entail just four hydrophobic amino acids: ILE, LEU, PHE, and TYR. LEU and ILE have a linear side chain with the carbon atom being the most protruding (see Figure 5) and are inter-medium in size (see Figure 6). PHE and TYR both have an aromatic ring consisting of C atoms, the one difference being that TYR has an -OH hydroxylic group attached to the ring. This makes the O atom the most protruding heavy atom for TYR (Figure 5). TYR, while still being overall hydrophobic, is larger and more water soluble than PHE, because of the -OH hydroxylic group. The R_max_ values of ILE, LEU, PHE, and TYR are 3.73 Å, 3.90 Å, 5.12 Å, and 6.45 Å, respectively.

**Figure 8:**
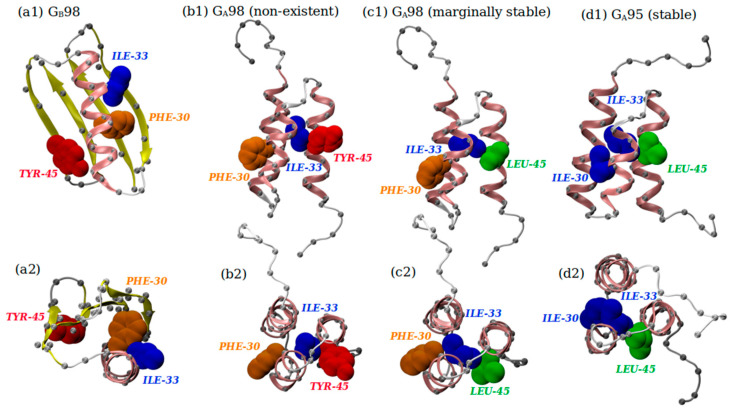
Side and top views of the folds adopted by highly similar amino acid sequences shown in Table 4. The G_A_ sequences adopt the topology of a three-helix bundle (3α-fold), while the G_B_ sequence adopts a α+4β fold. In all panels, the pink ribbons denote the portions of a chain that adopt the α-helical conformation, while the yellow ribbons form β-strands. Parts of a backbone that are not part of the secondary structure are shown in light gray. The darker gray spheres represent the positions of C_α_ atoms, whose radius is only 30% of the van der Waals radius of C atom, for ease of visibility. On the other hand, the heavy side chain atoms of the key amino acids responsible for changes in protein function and stability are assigned the van der Waals radii of the constituent atom types. Heavy atoms of ILE residues are shown in blue, LEU in green, TYR in red, and PHE in orange color. Panels (**a1**,**a2**) show the side and top views, respectively, of the α+4β topology of Protein G (G_B_98 sequence). Panels (**b1**,**b2**) represent side and top views of a ‘non-existent’ 3α fold for the same sequence as in Panels (**a1**,**a2**). Panels (**c1**,**c2**) represent the side and top views of the marginally stable G_A_98 sequence, whereas Panels (**d1**,**d2**) show the side and top views of the stable G_A_95 sequence. This stability is acquired by a single mutation from PHE to ILE at position 30, see Table 4.

Figure 8 shows three distinct sequences (shown in Table 4) (the sequences in Panels a and b are the same) along with two views (side and top views labeled 1 and 2) of three putative native state folds (the folds in Panels b and c are the same). We begin with Panels a1 and a2, which show the native state fold (α+4β topology) of Protein G. Panels b1 and b2 show a putative alternative fold (which is not realized experimentally) of the same sequence but with a 3α-topology. The 3α fold topology is not realized because of the TYR residue at position 45. To avert steric clashes, it is somewhat exposed to the water by pointing toward the protein exterior. The imperfectly fitting TYR residue also induces the non-ideal protrusion of ILE at position 33 that now less effectively fills the space in the protein interior. These insights are obtained primarily from the useful software package SCWRL4 [55] that determines the statistically most plausible side chain orientations that avert steric clashes.

The single mutation of TYR in position 45 to LEU leads to the remarkable fold switching from the α+4β topology to 3α-topology (Panels c1 and c2, see also Table 4). The geometrical distinction between TYR and ILE is in their disparate values of R_max_. One additional mutation, PHE at position 30 in the marginally stable 3α fold (G_A_98 sequence shown in panels c1 and c2 of Figure 8) into ILE, leads to a significantly increased stability of the three-helix bundle [47]. However, the snugger fit of ILE-30 in the hydrophobic core and its nestling with ILE-33 (see panels d1 and d2 of Figure 8) promote stability. In the interior of the α+4β fold, between the helix and the sheet (see Panels a1 and a2 of Figure 8), PHE-30 and TYR-54, hydrophobic amino acids with large side chains, play the critical role of filling the space.

## 4. Conclusions

We have presented the results of analyses of the behavior of side chains in experimentally determined native structures of over 4000 proteins. Our model is simplified, in the spirit of physics, and treats the protein backbone as a chain of C_α_ atoms. Only the heavy atoms of side chains are considered in our study. To have unbiased standardized results, which allows for variation in pseudo-bond lengths, we employ a backbone Frenet frame for our analysis.

We have considered several attributes of these side chains. We began with a proxy of structural chemistry by merely considering the constituent heavy atoms in the side chain, the identity of the most protruding atom, and the topology of the side chain (linear or ring) to divide the twenty amino acids into 14 groups. Remarkably, our rudimentary analysis is consistent with careful earlier studies resulting in the development of the much-used IMGT classification [54]. 

We then turned to the geometry of protrusion and found simplicity in that most side chains lie predominantly in the negative-normal–binormal plane. We went on to analyze the geometry and magnitude of protrusion of the amino acids. Our results show a rich range of behaviors of the side chains in terms of chemistry and geometry. There is a continuum of behaviors with an amino acid for every season.

We characterize the geometry by the protrusion of the farthest heavy atom from the C_α_ atom of the backbone. This protrusion has two main features: the distance of protrusion and the direction of protrusion. We characterize the latter using a novel Frenet coordinate system that can be applied to all amino acids. Our main contribution is a full description of the geometry of side chains within their native state structures. We correlate the geometry with secondary structure propensity and discuss in parallel the chemical nature of the amino acids.

## Figures and Tables

**Table 1:** Total number and relative frequency of twenty amino acid types in our data set comprising over 4000 protein native state structures, shown from the most abundant leucine (LEU) to the least abundant cysteine (CYS), along with the number of twenty amino acids in different protein contexts: helical ‘α’, strand ‘β’, and ‘loop’. Percentages shown in parenthesis are the frequencies with which each amino acid type is found in the respective protein context: helical ‘α’, strand ‘β’, and ‘loop’. GLY and PRO are the two amino acid types clearly distinct from others in that they strongly prefer the ‘loop’ environment (>70% of cases). ASN, ASP, SER, HIS, and THR prefer ‘loops’ as well, although more moderately (~50% of cases). Other amino acids are typically found in all environments, with occasional weak preference for ‘α’ or ‘β’.

Type	Total Number	Frequency [%]	α	β	Loop
LEU	84,916	8.85	36,154 (~43%)	21,387 (~25%)	27,375 (~32%)
ALA	82,208	8.57	38,896 (~47%)	13,583 (~17%)	29,729 (~36%)
GLY	76,284	7.95	10,839 (~14%)	10,883 (~14%)	54,562 (~72%)
VAL	69,481	7.24	20,194 (~29%)	29,569 (~43%)	19,718 (~28%)
GLU	61,780	6.44	28,135 (~45%)	9678 (~16%)	23,967 (~39%)
ASP	57,111	5.95	15,259 (~27%)	6795 (~12%)	35,057 (~61%)
SER	56,318	5.87	13,965 (~25%)	10,649 (~19%)	31,704 (~56%)
ILE	54,043	5.63	18,561 (~34%)	20,635 (~38%)	14,847 (~28%)
LYS	53,739	5.60	20,349 (~38%)	9605 (~18%)	23,785 (~44%)
THR	53,588	5.58	13,129 (~24%)	14,272 (~27%)	26,187 (~49%)
ARG	46,176	4.81	18,251 (~40%)	9217 (~20%)	18,708 (~40%)
PRO	44,397	4.63	6396 (~15%)	4148 (~9%)	33,853 (~76%)
ASN	42,128	4.39	9757 (~23%)	5804 (~14%)	26,567 (~63%)
PHE	38,853	4.05	12,348 (~32%)	12,184 (~31%)	14,321 (~37%)
TYR	34,685	3.61	10,506 (~30%)	10,825 (~31%)	13,354 (~39%)
GLN	34,361	3.58	14,372 (~42%)	5870 (~17%)	14,119 (~41%)
HIS	22,392	2.33	6261 (~28%)	4897 (~22%)	11,234 (~50%)
MET	19,524	2.03	8273 (~42%)	4513 (~23%)	6738 (~35%)
TRP	14,579	1.52	4698 (~32%)	4205 (~29%)	5676 (~39%)
CYS	13,128	1.37	3469 (~26%)	3656 (~28%)	6003 (~46%)

**Table 2:** Classification of amino acids into 14 groups, based on the side chain topology, the type of atoms it contains, and the type of the atom that is maximally protruding from the corresponding C_α_ atom.

Group I	PRO	Ring connects back to the backbone
Group II	ALA, ILE, LEU, VAL	Linear (C); C: max
Group III	PHE	Ring (C); C: max
Group IV	TRP	Ring (C, N); C: max
Group V	TYR	Ring (C, O); O: max
Group VI	ARG, LYS	Linear (C, N); N: max
Group VII	HIS	Ring (C, N); N: max
Group VIII	ASP, GLU	Linear (C, O, O); O: max
Group IX	ASN, GLN	Linear (C, N, O); N: max
Group X	SER	Linear (C, O); O: max
Group XI	THR	Linear (C, O); C: max
Group XII	CYS	Linear (C, S); S: max
Group XIII	MET	Linear (C, S); C: max
Group XIV	GLY	No heavy atoms

**Table 3:** Statistics of values of the angle ɛ between the projection of the most protruding vector in the anti-normal–binormal plane with the anti-normal direction. The positions of the most frequently observed value (mode) or modes (when there are more than one mode) are presented. The mean values and standard deviations of the angles ɛ_α_, ɛ_β_, and ɛ_loop_ characterizing the geometry of protrusion in three different contexts: α, β, and loop are also presented.

Type	ɛ_α_ Mode [°]	ɛ_α_ Mean [°]	ɛ_β_ Mode [°]	ɛ_β_ Mean [°]	ɛ_loop_ Mode [°]	ɛ_loop_ Mean [°]
PRO	105	104.9 ± 5.5	77	74.8 ± 13.1	73, 108	83.2 ± 21.1
ALA	50	50.0 ± 2.3	25	28.2 ± 7.3	30, 48	37.7 ± 10.4
ILE	45	37.2 ±15.9	12	20.0 ± 14.6	12, 53	29.3 ± 20.3
LEU	43	40.8 ± 5.8	16	19.4 ± 9.7	18, 38	27.9 ± 12.3
VAL	24	32.7 ± 15.5	5	16.3 ± 20.7	7, 23	29.8 ± 26.3
PHE	3	14.1 ± 14.6	3	24.5 ± 28.8	3	21.1 ± 25.2
TRP	18	30.5 ± 24.4	32	36.7 ± 23.4	30	39.9 ± 30.2
TYR	0	14.1 ± 17.1	4	25.6 ± 28.6	4	24.1 ± 27.6
ARG	30, 70	38.6 ± 24.1	2	23.9 ± 20.5	3	29.9 ± 23.5
LYS	38	31.1 ± 16.5	7	20.3 ± 16.2	12	27.8 ± 19.8
HIS	14	24.8 ± 18.4	5	19.6 ± 24.3	0	26.9 ± 27.7
ASP	42	42.2 ± 10.5	14	19.8 ± 16.2	10, 40, 60	34.9 ± 21.0
GLU	3, 35	29.3 ± 17.4	0	18.8 ± 17.4	3	29.9 ± 23.3
ASN	43	40.4 ± 11.0	15	22.3 ± 16.5	18, 37, 57	34.5 ± 19.5
GLN	0, 29	28.4 ± 17.1	0	20.7 ± 17.6	0	27.7 ± 20.9
SER	25, 38, 78	49.6 ± 22.9	3, 58	30.5 ± 27.0	10, 77	48.6 ± 28.3
THR	23	26.9 ± 9.3	5	16.4 ± 20.2	17	24.6 ± 16.8
CYS	32	32.9 ± 13.6	0	18.7 ± 24.8	3	29.2 ± 27.0
MET	0	28.7 ± 21.1	0	24.9 ± 17.7	0	24.5 ± 19.3

**Table 4:** Sequence alignment (of length 56) of the α+4β G_B_98 protein and two 3α G_A_ proteins in the one-letter amino acid code. The unique amino acid difference between the G_B_98 and G_A_98 protein sequences is at position 45 and denoted in bold. TYR (Y) in the G_B_98 sequence is replaced by LEU (L) in the G_A_98 sequence. The two 3α G_A_ protein sequences, G_A_98 and G_A_95, also differ by a single amino acid. PHE (F) at position 30 in the marginally stable G_A_98 sequence is changed (denoted by bold) to ILE (I) in the stable G_A_95 sequence.

Position	1 10 20 30 40 50
G_B_98	TTYKLILNLKQAKEEAIKELVDAGTAEKY**F**KLIANAKTVEGVWT**Y**KDEIKTFTVTE
G_A_98	TTYKLILNLKQAKEEAIKELVDAGTAEKY**F**KLIANAKTVEGVWT**L**KDEIKTFTVTE
G_A_95	TTYKLILNLKQAKEEAIKELVDAGTAEKY**I**KLIANAKTVEGVWT**L**KDEIKTFTVTE

## Data Availability

The raw data supporting the conclusions of this article will be made available by the corresponding author on request.

## Supplementary Materials

The following supporting information can be downloaded at: https://www.mdpi.com/article/10.3390/biom14070805/s1, Table S1: Statistics of the protrusion for all amino acids in our data set, as well as for the nineteen amino acids separately.
