# Supplementary material for: Amino-Acid Characteristics in Protein Native State Structures
Source: Biomolecules. 2024 Jul 7;14(7):805. doi: 10.3390/biom14070805 (PMC11274641; doi:10.3390/biom14070805)
Supplement: Supplementary file 1 [file biomolecules-14-00805-s001.zip › biomolecules-3060083-supplementary.pdf]

Supplementary Information for the manuscript: “Amino acid characteristics in protein native state structures”

Tatjana Škrbić, Achille Giacometti, Trinh X. Hoang, Amos Maritan, and Jayanth R. Banavar

Type	$\langle R_{max} \rangle$[Å]	R_{eff} [Å]	$R_{eff}/\langle R_{max} \rangle$	u_{AN}	u_T	u_B	$\langle \cos \theta \rangle$
**All**	**3.81**	**2.99**	**0.78**	**0.89**	**-0.04**	**-0.45**	**0.79**
**PRO**	2.43	2.31	0.95	0.02	-0.77	-0.64	0.95
**ALA**	1.53	1.49	0.98	0.74	0.14	-0.66	0.98
**ILE**	3.73	3.29	0.88	0.89	-0.20	-0.41	0.88
**LEU**	3.90	3.64	0.94	0.86	0.01	-0.52	0.93
**VAL**	2.54	2.09	0.82	0.88	0.24	-0.41	0.82
**PHE**	5.12	3.56	0.69	0.98	-0.09	-0.20	0.69
**TRP**	6.13	3.90	0.64	0.98	0.11	-0.16	0.63
**TYR**	6.45	4.19	0.65	0.98	-0.11	-0.17	0.65
**ARG**	6.49	5.04	0.78	0.90	-0.01	-0.43	0.77
**LYS**	5.78	4.74	0.82	0.93	-0.02	-0.36	0.81
**HIS**	4.56	3.24	0.71	0.93	-0.11	-0.34	0.71
**ASP**	3.61	3.16	0.87	0.82	-0.01	-0.57	0.87
**GLU**	4.60	3.71	0.81	0.92	-0.05	-0.38	0.80
**ASN**	3.60	3.07	0.85	0.83	-0.13	-0.54	0.85
**GLN**	4.54	3.65	0.80	0.94	-0.08	-0.33	0.79
**SER**	2.43	2.00	0.83	0.68	0.14	-0.72	0.83
**THR**	2.53	2.13	0.84	0.92	0.06	-0.38	0.84
**CYS**	2.80	2.29	0.82	0.89	-0.06	-0.45	0.82
**MET**	4.54	3.76	0.83	0.95	0.01	-0.31	0.82
**GLY**	-	-	-	-	-	-	-

Table S1. Statistics of the protrusion for all amino acids in our data set, as well as for the nineteen amino acids separately.
